# Glycosphingolipid metabolism and its role in ageing and Parkinson’s disease

**DOI:** 10.1007/s10719-021-10023-x

**Published:** 2021-11-10

**Authors:** Kerri-Lee Wallom, María E. Fernández-Suárez, David A. Priestman, Danielle te Vruchte, Mylene Huebecker, Penelope J. Hallett, Ole Isacson, Frances M. Platt

**Affiliations:** 1 Department of Pharmacology, University of Oxford, Oxford, UK; 2 Institute of Innate Immunity, Biophysical Imaging, Medical Faculty, University of Bonn, Bonn, Germany; 3 Neuroregeneration Institute, McLean Hospital, Harvard Medical School, Belmont, MA 02478 USA

**Keywords:** Parkinson’s disease (PD), Glycosphingolipid (GSL), Gangliosides, Ageing, *GBA*, Lysosomal storage disease (LSD)

## Abstract

It is well established that lysosomal glucocerebrosidase gene (*GBA*) variants are a risk factor for Parkinson’s disease (PD), with increasing evidence suggesting a loss of function mechanism. One question raised by this genetic association is whether variants of genes involved in other aspects of sphingolipid metabolism are also associated with PD. Recent studies in sporadic PD have identified variants in multiple genes linked to diseases of glycosphingolipid (GSL) metabolism to be associated with PD. GSL biosynthesis is a complex pathway involving the coordinated action of multiple enzymes in the Golgi apparatus. GSL catabolism takes place in the lysosome and is dependent on the action of multiple acid hydrolases specific for certain substrates and glycan linkages. The finding that variants in multiple GSL catabolic genes are over-represented in PD in a heterozygous state highlights the importance of GSLs in the healthy brain and how lipid imbalances and lysosomal dysfunction are associated with normal ageing and neurodegenerative diseases. In this article we will explore the link between lysosomal storage disorders and PD, the GSL changes seen in both normal ageing, lysosomal storage disorders (LSDs) and PD and the mechanisms by which these changes can affect neurodegeneration.

## Introduction

**Fig. 1:**
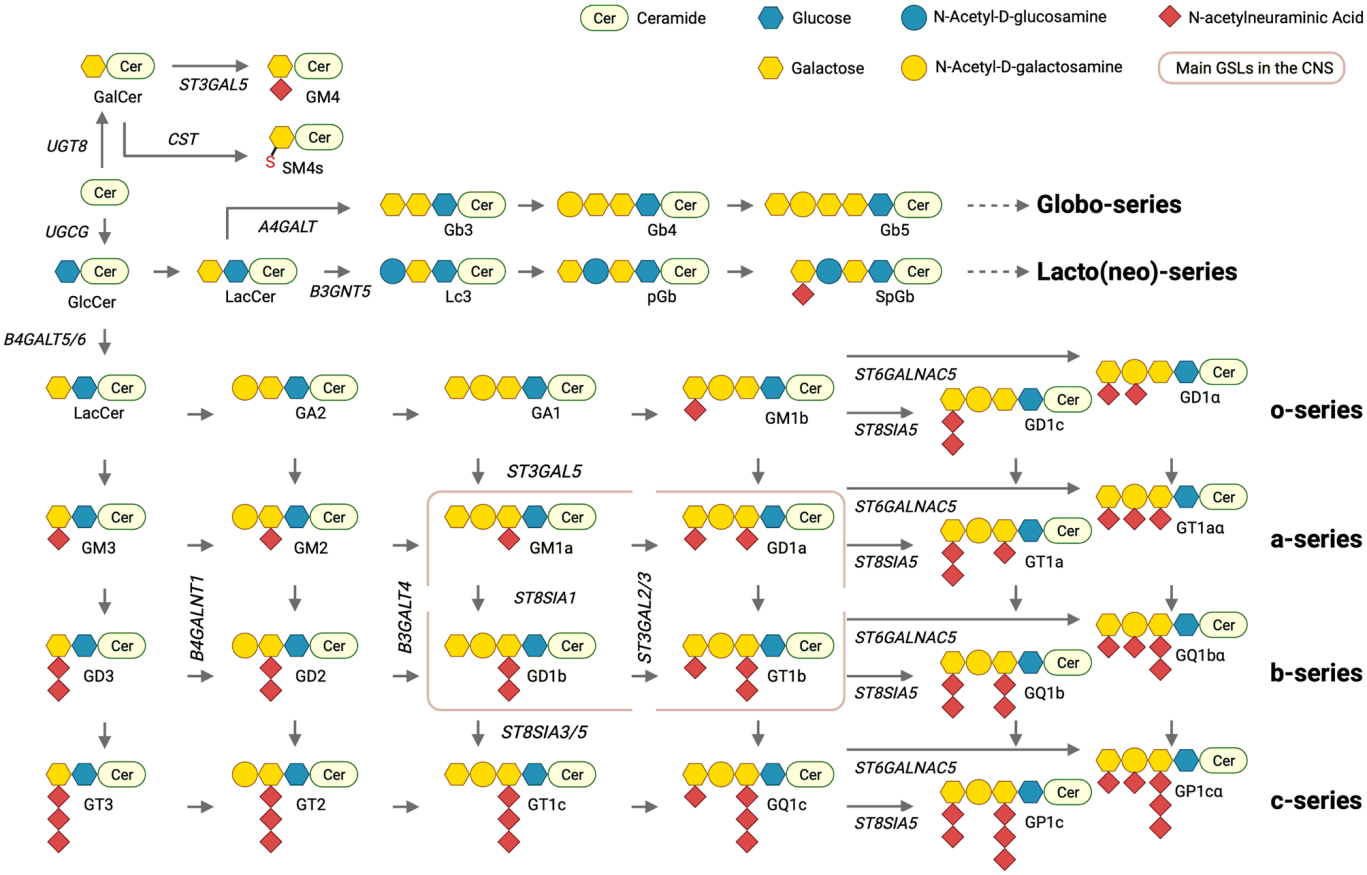
Simplified scheme of GSL biosynthesis. Major gangliosides expressed in the central nervous system (CNS) in adult mammalian brain are boxed in pink. Biosynthetic enzyme genes are indicated in blue. GSL names are abbreviated according to Svennerholm [123] and recommended by IUPAC [124]

**Fig. 2:**
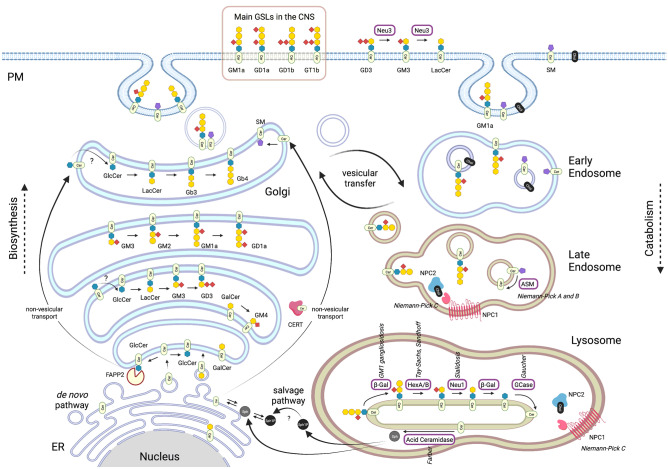
Metabolism and trafficking of GSLs. Ceramide is synthesised in the ER and transported to the Golgi by vesicular transport where it is converted to GlcCer. Ceramide can also be bound by CERT and transported by non-vesicular pathway to the late Golgi for the synthesis of SM [125]. Also, via a non-vesicular pathway, the transfer protein FAPP2 transports GlcCer from cis to trans golgi and couples it specifically to the synthesis of globosides [126]. GSLs are then carried by vesicular transport to the plasma membrane where they can be remodelled. Upon endocytosis GSLs are degraded into monosaccharides, free fatty acids, and sphingoid bases, which are recycled for sphingolipid synthesis by the salvage pathway (modified after [127]). ASM, acid sphingomyelinase; β-Gal, β-Galactosidase; Cer, ceramide; CERT, ceramide transfer protein; chol, cholesterol; ER, endoplasmic reticulum; FAPP2, phosphatidylinositol-four-phosphate adapter protein 2; GCase, glucocerebrosidase; GlcCer, glucosylceramide; Hex, hexosaminidase; LacCer, lactosylceramide; Neu, neuraminidase; PM, plasma membrane; SM, sphingomyelin; Sph, sphingosine; Sph1P, sphingosine-1-phosphate. *The figures were created with BioRender.com*

Glycosphingolipids (GSLs) are composed of a hydrophobic ceramide moiety linked to a hydrophilic glycan head group [1]. The complexity of GSLs is a result of the diversity of sugars in the head group (the monosaccharide type, number, and linkage) and heterogeneity in both the long-chain base and the fatty acyl moiety of the ceramide (chain length, hydroxylation, and saturation) [1] (Fig. 1). The biosynthesis of GSLs is also complex and begins in the ER with the generation of their precursor, ceramide, through the differential activities of multiple ceramide synthases (CERS) that generate ceramide backbones with different chain lengths [2, 3]. The first step in the biosynthesis of most GSLs is the transfer of glucose to the ceramide backbone on the outer leaflet of an early Golgi compartment, generating glucosylceramide (GlcCer) (Fig. 2). Neutral GSLs are synthesised in the trans Golgi, whereas gangliosides (GSLs containing charged sialic acids) are synthesised luminally in an early Golgi compartment [1]. The regulation of these two branches of GSL biosynthesis is regulated through vesicular and non-vesicular transfer, respectively. If GlcCer moves via vesicular transport through the Golgi stack, it is preferentially used to build gangliosides (starting with GM3). Non-vesicular transfer of GlcCer to late Golgi compartments is mediated by the action of the lipid transfer protein FAPP2 and leads to preferential production of globosides (starting with Gb3) [4]. GSLs have a stable cell-type specific expression pattern and mediate numerous biological functions, such as cell adhesion and migration, cell signalling, proliferation, endocytosis, intracellular transport, inflammation and apoptosis [5]. The biosynthesis, trafficking, and catabolism of GSLs is tightly regulated (Fig. 2).

Insights into GSL functions and metabolism have arisen in part from the study of inborn errors of metabolism in which genes encoding lysosomal enzymes involved in GSL catabolism are mutated. Typically, these diseases are inherited as autosomal recessive traits [6]. This family of rare diseases are termed lysosomal storage diseases (LSDs) and include Gaucher disease (*GBA* mutations) and Sandhoff disease (*HEXB* mutations). All sphingolipid lysosomal storage diseases are multimorbidity diseases and the majority have a neurodegenerative clinical course, emphasising the biological importance of GSLs in the brain. The mammalian brain GSL profile is dominated by gangliosides. The main four gangliosides, GM1a, GD1a, GD1b and GT1b make up > 90% of GSLs in the brain of all mammals, and reside primarily on the outer leaflet of the plasma membrane (PM) [7].

As mice engineered to be null for GlcCer synthase are embryonically lethal [8], it was hypothesised that there would be no human diseases arising from mutations in GSL biosynthetic genes as they would result in lethality during embryonic development. However, there are ultra-rare diseases of ganglioside biosynthesis that have been identified in Old Amish communities in the USA. For example, *B4GALNT1* variants, encoding GM2-synthase, lead to a lack of GM2 and higher GSLs (a-series), resulting in a complex form of hereditary spastic paraplegia, and in mice lead to parkinsonism [9]. Mutations in *ST3GAL5*, encoding GM3 synthase, which initiates the synthesis of all downstream cerebral gangliosides, result in an extremely severe epilepsy syndrome [10].

## The link between Gaucher disease and Parkinson’s disease

The first link found between an LSD and PD was the association with Gaucher Disease (GD). Initially clinicians observed an unusually high subset of GD patients as well as their relatives developing parkinsonism over time [11]. In 2009, a major worldwide multi-centre genetic study reported a significant association between mutations in the *GBA* gene, the genetic cause of GD, and sporadic PD [12]. Subsequently, it was established that 10–15% of those with heterozygous and homozygous *GBA* mutations develop PD, a 20-fold increased risk compared to non-carriers [13]. Furthermore, 5–15% of sporadic PD patients carry a *GBA* mutation and present earlier, meaning *GBA* is the highest genetic risk factor for developing PD and carrying this gene increases the rate of progression [14].

Homozygous mutations in the *GBA* gene, encoding the enzyme glucocerebrosidase (GCase), mean the enzyme is unable to degrade its GSL substrates GlcCer and glucosylsphingosine (GlcSph) [15]. It has always been believed that heterozygosity was not associated with storage or pathology, however no systematic studies have been performed on Gaucher carriers as they age to determine if they are more susceptible to certain diseases.

The pathogenic mechanism linking *GBA* to PD is unclear, but several studies have linked it directly to alpha-synuclein. A key question is whether this association results from gain or loss of enzymatic function. The fact that most GCase mutations result in misfolded protein could theoretically lead to a gain of function, either directly by causing alpha-synuclein aggregation, or by causing ER stress or lysosomal/autophagosomal disruption [16]. Mutant GCase has been found in 75% of Lewy bodies in post-mortem *GBA*-PD brain which could suggest it enhances aggregation of alpha-synuclein, or alternatively is simply the cell trying to clear it [17]. Supporting the gain of function hypothesis, there are mutations that do not cause GD but do predispose to PD [18]. However, there are null mutations that also predispose to PD, thus suggesting the pathological mechanism is not solely due to gain of function.

There is increasing evidence that it is loss of function of GCase that results in alpha-synuclein aggregation. Mazzulli et al. found that the lipid substrate GlcCer was able to stabilise alpha-synuclein oligomeric intermediates in the lysosome, which led to further reduction in GBA activity, resulting in a self-propagating feedback loop, leading to neurodegeneration [19, 20]. GCase also binds alpha-synuclein in the lysosome, but this interaction is reduced with mutant GCase [20]. A recent study further supported the direct effect on alpha-synuclein, indicating that GCase is able to inhibit lipid-induced alpha-synuclein amyloid formation, and that there is competition between lipid and GCase for alpha-synuclein binding [21].

Rocha et al*.* found that sustained GCase inhibition (with the chemical inhibitor conduritol B epoxide (CBE)) induces alpha-synuclein aggregation, microglial and C1q activation in mouse substantia nigra, with GlcCer and GlcSph increases leading to neurodegeneration [22]. This accumulation is cell type specific: both PD brains and CBE treated mice have increased neutral lipid accumulation in dopaminergic neurons and microglia, whereas astrocytes have an overall reduced lipid load [23]. Although a recent study found that reduced GCase activity in mice did not result in alpha-synuclein aggregation alone, but when misfolded alpha-synuclein was present, GCase activity modulated neuronal susceptibility to pathology [24]. In an over-expressing alpha-synuclein mouse model, gene therapy with GCase prevented alpha-synuclein accumulation in the substantia nigra and striatum, and overexpression of GBA prevented alpha-synuclein-mediated dopamine neuron degeneration in rats [25]. Furthermore, in a mutant *GBA* mouse model, CNS expression of GCase alleviated GlcSph and alpha-synuclein accumulation and, importantly, reversed cognitive impairment [26]. This is supportive of loss of function being the main driver of alpha-synuclein aggregation and subsequent neurodegeneration.

Interestingly, GCase activity is also reduced in sporadic PD patients who do not have *GBA* mutations [27]. It has been reported that progressive decline of GCase also occurs in normal ageing, and results in an increase in glucosyl sphingosine (GlcSph) in the substantia nigra [28]. Elevated GlcCer has been observed in iPSC-derived dopaminergic neurons from *GBA* associated PD patients, but studies differ as to whether there is accumulation of GlcCer and GlcSph in the human PD brain [16, 28–30]. In one large study, no association was found between storage of GlcSph and the hyperechogenic area of the substantia nigra, a prodromal PD marker in *GBA* mutation carriers [31].

Some studies have associated ‘severe’ *GBA* mutations with a higher risk of PD in carriers [32, 33] as well as an earlier onset and more rapid cognitive decline [34]. In CSF of PD with *GBA* mutations, GCase activity was decreased with a concomitant increase in GlcCer, and total alpha-synuclein was lower [30, 34]. This difference was greater in those with ‘severe’ risk *GBA* variants than ‘mild’ or ‘low’ risk. However, evidence shows there is low penetrance of PD with *GBA* variants, suggesting the existence of modifier genes that determine risk for PD [35, 36]. A genome-wide association study (GWAS) in mice identified 17 putative modifier genes, the highest significance being those involved in neuronal excitability (*Adk, Dpp10, Ctnnd* and *Grin2b*), and secondly in endolysosomal function and neurodevelopment [36]. A recent large GWAS analysing PD associated genetic risk score, to detect genetic influences on GBA risk and age of onset, found the most significant contributors were genes implicated in lysosomal function, notably *SNCA* and *CTSB* [35]. Schierding et al*.* also found a number of common variants (non-coding snps) that regulate *GBA* expression and co-regulate modifier genes [37]. Such evidence provides a possible explanation for the variable phenotypes seen in GBA-linked PD.

Several other known genetic risk factors for PD are directly linked to GCase activity. LIMP-2 (GBA trafficking receptor that delivers the enzyme to the lysosome) is encoded by *SCARB2*, of which gene variants are associated with PD [38]. These variants were not associated with reduced GCase activity; however, activity was measured in dried blood spots so would not be picked up if the enzyme were mis-localised [39]. In a recent study, GCase was found to be reduced in both *GBA* and non-*GBA* PD fibroblasts [40]. The idiopathic PD patients were found to have reduced LIMP-2 protein levels, and this correlated with GCase activity. Similarly, variants in *ATP10B*, which encodes a late endo-lysosomal lipid flippase that translocates GlcCer to the cytosolic leaflet, has been associated with PD risk [41]. A number of studies have also linked progranulin (PGRN) with PD. Heterozygous *PGRN* mutations cause frontotemporal dementia with parkinsonism, and reduced progranulin levels are associated with severity of PD [42–44]. Progranulin is lysosomal and thought to bind GCase leading to reduced GCase activity [45].

## Links between PD and multiple LSDs

Although Gaucher disease is the most extensively researched LSD in the context of PD, a number of other LSDs have been associated with parkinsonism, alpha-synuclein accumulation and substantia nigra pathology. The burden of gene variants associated with lysosomal storage disorders in sporadic PD is very significant [46]. A large genetic association study looked at fifty-four LSD genes in the largest PD whole exome sequencing dataset available. Fifty six percent of PD patients had at least one putative damaging lysosomal storage disorder gene variant and twenty one percent of PD patients carried multiple alleles [46]. As well as those previously linked to PD, the study newly implicated *SLC17A5*, *ASAH1*, and *CTSD* causing Salla disease, Farber lipogranulomatosis, and a neuronal ceroid lipofuscinosis variant, respectively. *SLC17A5* encodes a lysosomal membrane transporter for sialic acid, *ASAH1* encodes acid ceramidase, which participates in ceramide metabolism, and *CTSD* encodes a lysosomal aspartyl proteinase that has been implicated in alpha-synuclein degradation [47]. The overall implication of LSD-associated genes in PD is significant and indicates a link between defective catabolism of GSLs and/or other lysosomal substrates in PD pathogenesis.

It is interesting to note that in addition to multiple GSL catabolic genes being over-represented in sporadic PD, heterozygous mutations were identified in *ST3GAL5*, encoding GM3 synthase, which catalyses the first step in ganglioside biosynthesis (Fig. 1). GM3 synthase is a sialyltransferase responsible for the generation of GM3 in the Golgi apparatus. Deficiency leads to loss of complex gangliosides, and accumulation of LacCer and neutral globosides [48], causing an early onset severe epilepsy syndrome [10, 46]. This further underlines the imbalance in ganglioside expression as a potential risk factor in PD.

Alpha-synuclein aggregation has been observed in several LSDs [49]. Increased prevalence of PD has been seen in late onset Fabry Disease patients [50], and the deficient enzyme in Fabry disease, alpha-galactosidase, was found to be reduced in PD leukocytes and PD brain [51, 52]. Significant correlation was seen between decreased alpha-galactosidase, increased Gb3 and pathological accumulation of alpha-synuclein [51, 52]. Krabbe disease, caused by mutations in galactocerebrosidase (*GALC*), results in accumulation of GalCer, which leads to a similar pattern of alpha-synuclein aggregation in the brain of the mouse model to that seen in the human PD brain [53]. GalCer forms hydrophilic clusters and binds the C-terminus of α-synuclein [54]. Furthermore, *GALC* gene therapy completely prevented alpha-synuclein aggregation in the Krabbe mouse model [54]. GalCer was higher in cerebral cortex of PD patients, and controls with heterozygous *GALC* mutations had evidence of alpha-synuclein pathology [55]. Sandhoff disease is caused by mutations in *HEXB* gene, resulting in deficiency in the activities of the enzyme Hexosaminidase A and B, and the accumulation of GM2 and GA2. Sandhoff disease brains have also shown evidence of alpha-synuclein aggregation [56]. Deletion of *HEXB* results in alpha-synuclein aggregation in mice [57], whereas upregulation of beta-hexosaminidase expression (by AAV gene therapy) and activity in an alpha-synuclein rat model prevents alpha-synuclein lipid association and protects dopaminergic neurons [58].

Several studies suggest a link between the *NPC1* gene mutations and PD. Defects in *NPC1* are responsible for 95% of clinical cases of the autosomal recessive LSD Niemann-Pick disease type C (NPC). NPC is characterized by lysosomal accumulation of lipids including cholesterol and GSLs, and by reduced lysosomal calcium levels [59]. Lipid accumulation and reduced calcium levels within the lysosome are also present in PD [60, 61]. Furthermore, aberrant phosphorylation of alpha-synuclein and Lewy bodies have been found in several brain regions of NPC patients [62, 63]. A growing number of reports have identified mutations in one allele of *NPC1* in patients diagnosed with PD, parkinsonism or atypical PD, such as progressive supranuclear palsy (PSP) or corticobasal degeneration (CBD) [46, 64–66]. Some of these studies showed common clinical manifestations between NPC and PD, such as supranuclear gaze palsy and dementia [66]. Moreover, a recent study showed subclinical deficits in cognition in *NPC1* carriers that correlated with impairment of cholinergic circuits [67]. NPC has not explicitly been identified in genetic association studies of large PD cohorts. Nonetheless, its involvement cannot be ruled out, especially for atypical forms of parkinsonism, including PSP/CBD, for which more studies are needed [66, 68].

Mutations in the acid sphingomyelin phosphodiesterase 1 (*SMPD1*) gene, which is responsible for Niemann-Pick type A/B, have also been associated with PD [69]. Among PD patients, reduced acid sphingomyelinase activity was associated with a 3.5- to 5.8-year earlier onset of PD [70]. Furthermore, *SMPD1* knockout and knockdown resulted in increased alpha-synuclein levels in dopaminergic cell models [70].

One form of the LSD neuronal ceroid lipofuscinosis (NCL), known as Batten disease, is caused by homozygous mutations in the *GRN* gene, which encodes progranulin (PGRN), a lysosomal glycoprotein. Heterozygous *GRN* mutations cause frontotemporal dementia with parkinsonism [42]. In PD, it has been demonstrated that reduced levels of progranulin are associated with disease severity [43, 44]. Progranulin gene delivery has been shown to protect dopaminergic neurons in a chemically induced (MPTP) mouse model of Parkinson's disease [71]. PGRN has been shown to interact with GCase, being essential for its activity, which may explain its role in PD [45]. *ATP13A2*, encoding a predominantly neuronal endolysosomal ATPase, is another gene associated with a different form of NCL [72]. Also called PARK9, autosomal recessive mutations in this ATPase are associated with an early onset PD known as Kufor-Rakeb syndrome [73].

The alpha-synuclein accumulation seen in LSDs and its reversibility by increasing the expression of the GSL related gene or enzyme suggests that proteinopathy might be preceded by GSL dysregulation/lysosomal dysregulation in the course of PD pathogenesis .

## GSLs and ageing

The biggest non-genetic risk factor for PD is ageing. There have been several studies reporting age-related changes in sphingolipids (as well as cholesterol, phosphoinositides and polyunsaturated fatty acids) in the brain (reviewed in [74, 75]). The associations seen between LSDs and PD led to more in-depth studies of lysosomal enzyme levels and GSL levels in the ageing and PD brain. During human ageing, it was found that GBA activity declined in post-mortem substantia nigra and putamen (Table 1) [60]. The lysosomal enzyme levels of GBA as well as several other lysosomal enzymes (alpha-galactosidase, beta-hexosaminidase, beta-galactosidase and neuraminidase) (see Table 2) were reduced even further in PD patients, and the GSL substrate GlcCer progressively accumulated with age (Table 4) [60]. PD patients had significantly higher GlcCer levels in the substantia nigra compared to age-matched controls. However, the level of ganglioside GM1a in the substantia nigra declined with age in both the human control subjects and PD patients (Table 3 and 4) [60]. In PD patients all the main brain gangliosides (GM1a, GD1a, GD1b and GT1b) were significantly reduced compared to control subjects (Table 4) [60]. A reduction in gangliosides was also seen in CSF and plasma from PD patients compared to age-matched controls (Table 4), as well as in prodromal RBD (rapid eye movement sleep behaviour disorder) patients [60].

**Table 1:** Enzyme activity changes in ageing mouse [76] and human brain [60]. Arrows indicate significance *p* = < 0.05

	glucocerebrosidase (GBA)	β-glucosidase (GBA2)	α-galactosidase	β-hexosaminidase	β-galactosidase	neuraminidase
Mouse	↓	↓	⎻	⎻	↓	↑
Human Controls Substantia Nigra	↓	⎻	⎻	⎻	↓	⎻
Human PD Substantia Nigra	↓	⎻	⎻	↓	⎻	⎻

**Table 2:** Enzyme activity changes in PD vs age matched controls [60]. Arrows indicate significance *p* = < 0.05

	glucocerebrosidase (GBA)	β-glucosidase (GBA2)	α-galactosidase	β-hexosaminidase	β-galactosidase	neuraminidase
Substantia Nigra	↓	↓	↓	↓	↓	↓

**Table 3:** GSLs level changes in ageing mouse [76] and human brain [60]. Arrows indicate significance *p* = < 0.05

	Total GSLs	Total gangliosides	GlcCer	GlcSph	LacCer	GM1a	GD1a	GD1b	GT1b
Mouse	↑		↑	↑	↑	↑	↓	↓	↓
Human Controls Substantia Nigra	⎻	⎻	⎻	⎻	⎻	↓	⎻	⎻	⎻
Human PD Substantia Nigra	↑	↓	↑	⎻	⎻	↓	⎻	↓	⎻

**Table 4:** GSLs level changes in PD vs age matched controls [60]. Arrows indicate significance *p* = < 0.05

	Total GSLs	Total gangliosides	GlcCer	GlcSph	LacCer	GM1a	GD1a	GD1b	GT1b	GM3	GM2	GD3	Gb3	Gb4
Substantia Nigra	↑	↓	↑	↑	⎻	↓	↓	↓	↓				⎻	
CSF		↓			↑	⎻	↓	↓	↓	↑	↓	↓		
Serum			⎻		⎻	↓	↓			⎻	⎻		⎻	⎻

These age-related GSL changes were also conserved in mice, with GCase reduction and GlcCer increase, and a reduction in gangliosides, with the notable exception of GM1a [76]. Interestingly, in the murine brain, GM1a increased with age, as opposed to decreasing in the human brain, perhaps due to a compensatory mechanism (Table 3) [76]. Although the mouse data is from whole brain homogenates and must be interpreted with caution, it may suggest an interesting difference between mouse and human brain GSL biochemistry. A similar bypass was seen in the engineered *Hexa*^*−/−*^ Tay-Sachs disease mouse [77]. The mouse as a model organism only develops a Tay-Sachs disease phenotype when the sialidase enzyme neuraminidase 3 (encoded by *Neu3*), which is thought to facilitate GM2 degradation through this bypass pathway, is knocked out, in combination with the primary *Hexa* gene [77]. Neuraminidase activity normally increases with age in the mouse and combined with HEXB activity can bypass the Tay-Sachs disease catabolic defect allowing the mouse to escape disease [76, 77].

## The relevance of GM1a to PD

The complex ganglioside GM1a is of known importance in PD (reviewed in detail in [78]). GM1a levels increase during neuronal development, regulating calcium flux across the nuclear membrane [79] and interacting with proteins such as NGF (nerve growth factor), BDNF (brain-derived neurotrophic factor) and GDNF (glial-derived neurotrophic factor) via Trk tyrosine kinases promoting signalling [80]. BDNF signalling is involved in neuritogenesis, differentiation and survival [81]. Decreased BDNF is seen in serum and brain in PD and correlates with degeneration of dopaminergic neurons [82, 83]. Failure of GDNF signalling has been associated with dopaminergic cell loss in PD and GDNF has been investigated as a PD therapy, though it was able to rescue neurons only in the early stages of degeneration in rats and has limited success in human PD patients [84–86]. Huebecker et al*.* found that GM1a decreases with age in the substantia nigra and putamen in humans, and this occurs to an even greater degree in the PD brain [60]. A reduction in GM1a levels was previously noted in the substantia nigra and occipital cortex from PD patients, and specifically in the nigral dopaminergic neurons compared to age-matched controls [87, 88]. A significant decrease in both *B3GALT4 (GM1 synthase)* and *ST3GAL2 (sialyltransferase-4)* gene expression, resulting in reduced GM1a expression, was shown in residual neuromelanin-containing cells in the substantia nigra of PD patients compared to age-matched controls [89].

Several in vitro cell and mouse models have implicated GM1a as important player in PD pathology. Inhibition of GM1 synthase in neurons in vitro using siRNA decreased GM1a levels and increased cell vulnerability to the neurotoxin MPTP [90]. *B4galnt1*^±^ mice have reduced GM1a and substantia nigra neuropathology as well as gastro, cardiac and cognitive symptoms [87, 88]. Treatment of *B4galnt1*^±^ mice with GM1a led to decreased alpha-synuclein and rescued the motor deficits [88]. Surprisingly, treatment with only the oligosaccharide portion of GM1a resulted in complete rescue of the *B4galnt1*^±^ mice, suggesting the oligosaccharide is the bioactive element responsible for neurotrophic function [91]. No differences in GM1a or gangliosides were seen in the brain, therefore the treatment did not alter the GSL pathway [91]. It should be noted that *B4galnt1*^±^ mice present with a reduction in all complex gangliosides in the brain, but GM1a is only slightly reduced, whereas GD1a is further reduced, and GD3 and GD1b are significantly increased [9] (see Fig. 2). A GD3 synthase (*St8sia1*) (b-series) lentiviral shRNA KO mouse model demonstrated protection against MPTP-induced PD, had increased GM1a (and GD1b) in the brain, and reduced nigrostriatal damage, bradykinesia, and fine-motor-skill deficits [92]. One study which aimed to increase endogenous GM1a, injected *Vibrio cholera* sialidase in a rodent PD model (MPTP)[93]. Although sialidase administration did not demonstrate as much benefit good efficacy as systemic GM1a administration on all aspects of pathology, it did show similar efficacy in sparing of the DA neurons and as such may be a promising route [93]. In a rat alpha-synuclein model GM1a administration reduced alpha-synuclein aggregation, protected against loss of substantia nigra dopamine neurons and striatal dopamine levels, and furthermore, delayed start of GM1a administration was able to reverse behavioural deficits [94].

After numerous smaller studies on the use of GM1a (administered via subcutaneous injection) clinical trials were initiated and demonstrated clinical efficacy in PD patients [95, 96]. GM1a administration was shown to be safe and resulted in a slower rate of progression of PD symptoms [96]. These promising results warrant further research into GSLs, including the oligosaccharide moieties, as neuroprotective therapies.

## Relevance of other gangliosides to PD

When discussing the mouse models used to investigate GM1a, such as *B4galnt1*^±^ and *St8sia1*^*−/−*^, it is important to understand that the GSL alterations are not confined only to GM1a. It is becoming apparent that the GSL landscape in the human brain is altered with ageing and in PD. In addition to reductions in GM1a; GD1a, GD1b and GT1b are also reduced in PD substantia nigra (Table 4) [60]. Another small study found a similar reduction in GD1a , GT1b, GD1b, and GM1a in the substantia nigra of PD patients, as well as an increase in GlcCer and sphingomyelin [97].

There is evidence that GD1a is also involved in neuronal development. GD1a injection into the striatum of MPTP-treated mice significantly increased striatal dopamine levels, to a greater extent than GM1a, in aged mice [98]. GD1a/GT1b play a role in myelination and axon-myelin stability, via interaction with myelin-associated glycoproteins (MAGs) [99, 100]. GQ1b has neurotrophic actions such as increasing neurite outgrowth, cell proliferation, and long-term potentiation, by regulating BDNF [101]. The neuraminidase inhibitor DANA increased GQ1b/GT1a expression in neuronal culture and was shown to regulate glutamate release [102]. The addition of endogenous GQ1b was shown to ameliorate cognitive impairments via BDNF in another neurodegenerative disease, Alzheimer’s (in a triple transgenic mouse model), and GQ1b increased BDNF expression more effectively than GM1a [103]. As well as a reduction of the complex brain gangliosides, GD3 is increased during both normal human ageing and in neurodegenerative disorders [104]. Interestingly, intranasal GD3 was shown to reduce alpha-synuclein in the A53T overexpressing mouse model in a similar fashion to GM1a [105]. However the addition of GM1a and GD3 had opposing effects on dopaminergic TH levels [105].

Neuraminidases (most likely neuraminidase 3) have the unique ability to re-model gangliosides at the plasma membrane meaning cells can adapt rapidly to stimuli and are not reliant solely on de novo biosynthesis. Neuraminidase activity actively regulates memory processing in rat hippocampus and enzymatic activity is increased by BDNF signalling [106]. Neuraminidase levels were also shown to decrease with age in mice and humans (substantia nigra), and therefore their ability to finely regulate the plasma membrane surface expression of GSLs in the brain may be restricted [60, 107]. The age-related reduction in enzymes involved in GSL metabolism, including GCase and neuraminidases, likely accounts for these changes in GSL composition in the ageing brain and may be relevant to PD risk.

## GSLs and neurodegeneration

The direct toxic effects of GlcCer and GlcSph remain elusive, particularly in GBA-linked PD. Although the author’s studies found a clear increase in GlcCer and GlcSph levels in the substantia nigra of PD patients [28, 60], previous studies have found no accumulation in the PD brain [29]. In CBE treated mice, there was a direct correlation between the amount of CBE injected and levels of accumulation of GlcCer and GlcSph [108]. Disease pathology, indicated by altered levels of pathological markers, depended on both the levels of accumulated lipids and the time at which their accumulation began. Both GlcCer and GlcSph have been reported to specifically promote the formation of oligomeric alpha-synuclein and thus mediate neuropathology in GBA-associated PD [109]. However, when the hyperechogenic area of the substantia nigra, a prodromal PD marker, was measured in a large cohort of GBA mutation carriers (n = 71) and patients with GD (n = 145) it did not correlate with levels of GlcSph [31].

The mechanism by which GM1a exerts protection from neurodegeneration is still uncertain. It has been postulated that it is involved in the degradation of alpha-synuclein via enhanced autophagosomal activity [110]. It has also been shown that alpha-synuclein binds GM1a rich domains in synaptic vesicle fusion [111]. GM1a acts as a plasma membrane anchor for alpha-synuclein, which adopts a stable, alpha helical structure when bound, but in the absence of GM1a, starts to form fibrils [112].

Impaired GSL metabolism can also affect a plethora of downstream cellular processes. GSL accumulation, as well as preventing normal lysosomal degradation, affects fundamental processes such as mitochondrial respiration and autophagy [113]. The most commonly used PD model relies on the neurotoxin MPTP that selectively affects the dopaminergic neurons of the substantia nigra. MPTP has been shown to cause lysosomal dysfunction and its effects to be age dependent [114]. Lysosomal disruption causes alpha-synuclein accumulation, and alpha-synuclein reciprocally causes lysosomal dysfunction. 

GD3 is reported to be involved in autophagosomal biogenesis [113] and has been shown to mediate mitochondrial apoptosis in many cell types [115]. Gangliosides, in particular GD3, can also induce apoptosis in astrocytes [116]. However, in a mouse model of Gaucher disease the absence of apoptotic cell death and caspase activation despite the onset of overt neurodegeneration implicated necroptosis rather than apoptosis [117]. Levels of RIPK1 and RIPK3 were elevated both in microglia and neurons correlating with neuroinflammation and neuronal cell death and RIPK3 deficiency resulted in increased survival [117].

Membrane contact sites (MCS), between organelles, and between organelles and the PM, are essential for signalling and metabolite exchange, and are composed of discreet microdomains of tightly regulated lipids including GSLs [118]. Failure to digest or process the GSLs in lysosomes alters the lipid composition and functional properties of MCS. It has been demonstrated that failure to degrade GM1a led to redistribution of GM1a to the PM and ER, as seen in the LSD GM1 gangliosidosis. GM1a accumulation at mitochondria:ER MCSs caused Ca^++^ dependent mitochondrial apoptosis [119] a possible cause of neuronal cell death and neurodegeneration [118]. *GBA*-PD patient-derived dopaminergic neurons exhibit prolonged mitochondrial:lysosomal contacts, resulting in disrupted mitochondrial distribution and function [120]. This was recapitulated with endogenous GlcCer treatment, and GBA-PD neuron tethering could be rescued by increasing GCase activity with a GCase modulator [120].

GSLs at the PM are tightly regulated in a cell-specific manner, and different cell types—neurons, astrocytes, and microglia—appear differentially affected by changes in GSLs [23]. Dopaminergic neurons of the substantia nigra appear to be particularly susceptible in PD and may reflect distinct GSL changes. Remodelling at the PM would likely disrupt neuronal PM interactions such as those of GM1a with BDNF, GDNF and NGF via Trk receptors [121], calcium channel signalling and synaptic transmission of dopamine and therefore negatively affect neuronal survival [78]. Interestingly, cell studies demonstrated that endogenous oligomeric GM1a administration was intercalated into the PM and did not enter the cell, confirming its neuroprotective action was at the PM [122]. The gangliosides play roles in neuronal homeostasis including myelination, axon formation, signalling and neurotransmitter release [80, 100, 101, 121, 122]. In the brain, GSLs are therefore not only involved in general lysosomal/autophagic cell functions and mitochondrial function but also at the plasma membrane in neuronal signalling and survival, and therefore changes in GSL levels increase the risk for PD. Although GM1a is of importance in neuronal function, and administration of GM1a has shown modest effects in the human disease, the restoration of normal neuronal GSL levels and normal GSL metabolism potentially may be of even greater benefit.

## Conclusion

The importance of altered GSL metabolism has become apparent in the study of normal ageing of the brain. Changes in the GSL ‘landscape’ with ageing (relative amounts and distribution) represents a risk factor for PD beyond *GBA* and may explain why so many LSD-causing gene variants are found over-represented in the heterozygous state in sporadic PD [46]. The precise mechanisms that underpin this increased risk are currently under investigation and may be multifactorial. GSLs are involved in mediating a plethora of cellular functions such as trafficking, autophagy, and signalling. Their biosynthesis and expression are highly cell type specific. As evidenced by the LSDs, the CNS is particularly vulnerable to lysosomal and GSL perturbations. In neurodegenerative diseases such as PD the brain shows age-related regional patterns of degeneration. Normal age-related GSL changes, possibly due to alterations in GSL metabolising enzyme activity, exacerbated by any one of a number of genetic or environmental modifiers, could lead to neurodegeneration and therefore PD. With greater knowledge of these risk factors, it may be possible to identify patients at greater risk of PD and target novel clinical intervention points, some of which may relate to restoring GSL homeostasis.

## Data Availability

Not applicable.

## Abbreviations

BDNF: Brain-derived neurotrophic factor
CBD: Corticobasal degeneration
CERS: Ceramide synthases
CNS: Central nervous system
CBE: Conduritol B epoxide
*GALC*: Galactocerebrosidase
GCase: Glucocerebrosidase
GDNF: Glial-derived neurotrophic factor
GlcCer: GlucosylCeramide
GlcSph: GlucosylSphingosine
GSL: Glycosphingolipid
LSD: Lysosomal Storage Disease
MCS: Membrane contact sites
NCL: Neuronal ceroid lipofuscinosis
NGF: Nerve growth factor
NPC: Niemann-Pick Disease
PD: Parkinson’s disease
PGRN: Progranulin
PSP: Progressive supranuclear palsy
